# A qualitative study of healthcare providers’ attitudes toward assisted partner notification for people with HIV in Indonesia

**DOI:** 10.1186/s12913-022-08943-x

**Published:** 2023-01-24

**Authors:** Judith A. Levy, Valerie A. Earnshaw, Ariesta Milanti, Agung Waluyo, Gabriel J. Culbert

**Affiliations:** 1 Health Policy & Administration, School of Public Health, University of Illinois Chicago, Chicago, IL USA; 2 Human Development and Family Sciences, College of Education and Human Development, University of Delaware, Newark, DE USA; 3 The Nethersole School of Nursing, The Chinese University of Hong Kong, Shatin, New Territories, Hong Kong SAR, China; 4 Faculty of Nursing, Universitas Indonesia, Kota Depok, Jawa Barat Indonesia; 5 Department of Population Health Nursing Science, College of Nursing, University of Illinois Chicago, Chicago, IL USA

**Keywords:** Contact tracing, HIV testing, Indonesia, Partner notification, Prisoner

## Abstract

**Background:**

Assisted partner notification (APN) is recommended as a public health strategy to increase HIV testing in people exposed to HIV. Yet its adoption in many countries remains at an early stage. This qualitative study sought the opinions of HIV health service providers regarding the appropriateness and feasibility of implementing APN in Indonesia where such services are on the cusp of adoption.

**Methods:**

Four focus group discussions totaling 40 health service providers were held in Jakarta, Indonesia to consider APN as an innovative concept and to share their reactions regarding its potential implementation in Indonesia. Voice-recorded discussions were conducted in Bahasa, transcribed verbatim, and analyzed.

**Results:**

Participants recognized APN’s potential in contacting and informing the partners of HIV-positive clients of possible viral exposure. They also perceived APN’s value as a client-driven service permitting clients to select which of three partner notification methods would work best for them across differing partner relationships and settings. Nonetheless, participants also identified personal and health system challenges that could impede successful APN adoption including medical and human resource limitations, the need for specialized APN training, ethical and equity considerations, and lack of sufficient clarity concerning laws and government policies regulating 3^rd^ party disclosures. They also pointed to the job-overload, stress, personal discomfort, and the ethical uncertainty that providers might experience in delivering APN.

**Conclusion:**

Overall**,** providers of HIV services embraced the concept of APN but forecast practical difficulties in key service areas where investments in resources and system change appeared necessary to ensure effective and equitable implementation.

**Supplementary Information:**

The online version contains supplementary material available at 10.1186/s12913-022-08943-x.

## Introduction

Over the last 20 years, considerable success has been achieved worldwide in the scale-up of HIV testing and referral to treatment for people who contract HIV. Nonetheless, of the approximately 38 million people estimated globally in 2020 to be living with HIV, about 16% (6 million) are thought to be unaware of having contracted the virus [1]. Closing this testing gap calls for effective new methods to reach this population. In recent years, assisted partner notification (APN) services have gained attention and increasing implementation as an innovative method to meet this challenge [2].

APN is a comprehensive, client-centered program to increase uptake of HIV testing, care, and treatment among partners of HIV-positive clients by notifying them of possible exposure to the virus and offering or linking them to testing [3]. In contrast to traditional public-health contact tracing that is provider led, APN allows clients who test HIV-positive to select voluntarily between three modes of disclosure. *Self-notification* refers to an agreement between the provider and client in which the latter chooses to self-tell partners about HIV exposure and then refer them for testing. This modality can include an agreement with the client that permits health care providers to inform partners of possible HIV exposure without naming the client if self-tell notifications are not completed by an agreed upon date. With *provider referral,* a health care provider with the permission of the client contacts partners to inform them of shared exposure and the need for testing without revealing who named them. With *dual referral*, a trained provider and client work as a team to inform the partner and encourage testing. Irrespective of which option the client chooses, APN’s goal is to inform the partner of possible exposure, offer or encourage HIV testing, and link those who test HIV-positive to HIV care and treatment [4].

A growing body of research testifies to the feasibility of implementing APN in a variety of settings and with different key populations [5–10]. Compelling evidence of its success comes from a meta-analysis of ten studies, including three individually randomized trials, showing that APN services increase the uptake of HIV testing services among exposed partners [8]. Additional research shows APN to be cost-effective in reducing HIV-related mortality and disability, while lending itself to task shifting [11]. APN may be especially effective in reaching first-time testers [12]. APN’s contact with HIV-positive individuals also can lead to reengagement in treatment among partners who know their status but have dropped out of care [13]. Based on this growing body of positive evidence, the World Health Organization endorses APN’s adoption along with recommendations to guide its implementation internationally [3, 4].

Despite its many advantages, adoption of APN is at an early stage in Indonesia. Questions remain as to how APN might be implemented within different social and epidemiological contexts while retaining the core components that make its services acceptable, ethical, and effective. A step toward achieving these goals is to consider in advance of its adoption how health care providers, clients, and stakeholders weigh the evidence for its implementation [14]. Formative research can help to identify components that need modification while possibly reducing future staff and organizational friction by identifying potential points of contention [15]. Such preliminary assessments also can usefully inform strategies to facilitate its acceptance among health care providers as future APN implementers.

### The current study: APN opportunities in Indonesia

This research reports the qualitative findings of a formative, multimethod study in Jakarta to assess the acceptability and feasibility of instituting APN as an innovative service new to Indonesia, a populous Southeast Asian country with a large and expanding HIV epidemic. Jakarta, its capital city, is home to an estimated 110,000 people with HIV [16]. HIV prevalence remains high among key populations, including an estimated one quarter of its population of men who have sex with men (MSM) [17]. Nonetheless, current epidemiologic models predict significant growth in the rate of future infections among spouses, casual sex partners, and women receiving antenatal services who are not members of key populations, but acquire HIV from a partner who is [16]. Such growth in HIV incidence is based on an expected increase in HIV testing among non-key populations [18], and also the fact that individuals of both sexes often are unaware of a partner’s sexual infidelity or substance use that places them both at risk [19, 20].

APN could help to counteract trends in new infections by linking persons with known HIV exposure to Indonesia’s existing continuum of publicly-funded HIV testing and treatment services, a plan that the government currently is considering [21]. Yet, Indonesian guidelines for partner contact tracing, much less APN, do not yet exist beyond recommending that spouses of HIV-positive clients get tested but without specifying how or when this should occur [21]. Meanwhile, the healthcare system already faces major challenges in providing basic services to people with HIV along with a critical shortage of HIV-trained staff [22]. Studies also describe increasing levels of HIV stigma and social hostility toward Lesbian, gay, bisexual, and transgender (LGBT) populations in Indonesia, including discrimination by health care providers [23–25]. Violations in Indonesian women’s reproductive right to HIV testing information, informed decision-making, privacy and confidentiality also have been found that impede their access to HIV testing and services [20]. Given these potential challenges to APN’s successful implementation at both the community and clinic level, a closer examination is warranted into how core HIV service providers view the future integration of APN into the services that they deliver.

To this end, four focus groups consisting of 40 HIV health care providers in total were convened in Jakarta to gauge their expert opinion of APN as a new approach to contact tracing and partner testing. Would they see APN services as relevant and possibly embrace the concept in their own work? How easy or difficult did they perceive it would be to implement APN services within the context of their organizations and with clients whom they serve? Finally, we wanted to gain insights into aspects or components of APN that might prove especially challenging for health providers to deliver and the possible facilitators and barriers at the personal and system level to its successful implementation. Findings from this study hold the potential to help guide the implementation of APN services in Indonesia and possibly other countries that are considering adopting it.

## Methods

Four voice-recorded focus group discussions were held with a total convenience sample of 40 nurses, physicians, and peer counselors serving HIV key populations in Jakarta, Indonesia. These occupations were sampled for their key role in delivering HIV prevention services in Indonesia [26, 27] and in other APN studies [8]. Meta-analysis research has shown that a group size of 4–8 members is sufficient to reach data saturation, the point at which the same themes and information tend to repeatedly emerge and the returns on holding additional group sessions diminish [28]. Discussions focused on the providers’ experiences with delivering HIV counseling and testing services and their perception of the facilitators and challenges to implementing APN services within the context of the current Indonesia health care system and their own organizations and practice settings.

### Recruitment and eligibility

Forty focus group participants were recruited through their professional networks via email, social media, and in-person. Contact points included a listserv for nurses in West Java and two large public HIV referral hospitals, four community health centers, and three non-governmental organizations (NGOs) serving key HIV populations in Jakarta. Eligible participants were: ≥ 18 years of age and employed in clinics, hospitals, and organizations serving key populations and people with HIV. The study was approved by Institutional Review Boards in the U.S. and Indonesia. Informed consent was obtained from participants. Each received $20 USD compensation for their time and transportation costs.

### Data collection and analyses

A short, written questionnaire collected participant demographic and occupational information. Group discussions were facilitated by a masters-prepared nurse researcher fluent in Bahasa (the Indonesian language) and familiar with APN, but without prior APN occupational experience. After explaining the research purpose, the facilitator provided an overview to APN to assure that everyone was familiar with the topic prior to discussion. A discussion guide in Bahasa (Additional file 1) was developed for the study to steer participants toward topics of interest. Each approximately 90-min discussion was voice recorded, transcribed verbatim in Bahasa, translated into English, and then back translated by a second researcher for accuracy and quality. Four members of the research team separately coded the data, and the independent analyses were compared for shared insight and coding consensus. The Promoting Action on Research Implementation in Health Sciences (PARIHS) framework [29] guided the choice of focus group questions. This conceptual model calls for collecting and evaluating participants’ assessment of scientific or experiential *evidence* in support of or against adopting a new health technology and the identification of key *contextual factors* possibly influencing its *implementation* at the provider, health system, and societal levels. Grounded Theory [30] was used to openly code and inductively identify emerging themes and reoccurring patterns of opinion and behavior within the data.

## Results

As shown in Table 1, the 4 focus groups were composed of peer educators and HIV test counselors working in community-based HIV service organizations (*n* = 24), nurses (*n* = 17), and physicians (*n* = 2). On average, participants had more than 10 years of occupational experience serving key populations and people with HIV. About half were university graduates (n = 23). Most (93%) had experience with HIV test counseling. With one exception, all were familiar with the concept of partner notification and public health contract tracing. Many had experience informing someone who had tested HIV-positive, and many also had experience in contacting members of key populations for HIV testing. None, however, had delivered APN with its service choice for clients between anonymous notification or self-disclosure per partner. Also, none had experience with following a chain of notification referrals or in offering point of contact HIV testing within the community as part of anonymous notification. Participants drew upon their personal experience in delivering HIV services, including individual and couple counseling, and their work in general to assess the facilitators, barriers, rewards, and drawbacks to implementing APN nationally and in local health care facilities.

**Table 1:** Participant characteristics [*n* = 40]

Characteristic	n (%)
**Age in years** (mean ± SD)	38.6 ± 7.7
**Female**	24 (57.1)
**Education**	
High school	10 (25.0)
Diploma	7 (17.5)
Bachelor’s degree	21 (52.5)
Post-baccalaureate	2 (5.0)
**Occupation**	
Community health worker	21 (52.5)
Nurse	17 (42.5)
Physician	2 (5.0)
**Occupational experience** (mean ± SD)	12.4 ± 7.3
Have you had any experience delivering health services to people with HIV?	
none	0 (0)
a little	6 (15.0)
a lot	34 (85.0)
Have you had any experience delivering health services to prison inmates?	
none	11 (27.5)
a little	17 (42.5)
a lot	12 (30.0)
How often do you conduct HIV test counseling?	
never	3 (7.5)
seldom	11 (27.5)
often	26 (65.0)
Have you ever informed someone of having tested positive for HIV?	
never	9 (22.5)
a few times	11 (27.5)
many times	20 (50.0)
Have you ever contacted someone about possible exposure to HIV?	
never	8 (20.0)
a few times	13 (32.5)
many times	19 (47.5)
How much do you know about HIV partner notification?	
nothing	1 (2.5)
a little	22 (55.0)
a lot	17 (42.5)

*Legend: SD* Standard deviation

### Perceptions of assisted partner notification

While not always fully familiar with the concept of APN, participants could envision its potential and were intrigued at its novelty and possible utility. As one peer educator exclaimed:

Helping clients to notify their exes and asking them, “Do you have any sexual partners? Have you ever had sex?” – it never happens. That is beyond anything that I have ever thought about, but it is great to think of reaching out to people, tracing people.

Another participant remarked that while the idea of contacting exposed partners was not new, working collaboratively with the client to identify and contact them was novel. He reminisced that “in the old days, we used to disregard who’s the partner.” He went on to explain that in his view “case findings would be much higher if patients directly disclose who their partners are.”

Participants said that clients whom they counseled often reported practical or emotional difficulties in informing their partners of an HIV diagnosis. They perceived that confidential notification services could relieve reluctant clients of the burden of telling their partners themselves. One nurse recounted the dilemmas of a male client who had yet to tell his wife:

The husband was incarcerated. He was concerned as his wife wanted to have another child. [Yet] he had not opened up to his wife about his [HIV] status. [He asked] “What should I do? I am afraid that my wife might get infected.

For this client, disclosing that he was HIV-positive could end his wife’s dreams of safely conceiving another child and might open him to unwanted questions as to how he had contracted the virus. Participants agreed that APN services could prove useful in helping clients to resolve such dilemmas.

### Persuading clients to name and notify partners

While the idea of offering APN services was met with enthusiasm, delivering them was seen as needing careful presentation and management. Clients whom they serve frequently are reluctant to notify a partner, and their reactions to being asked to do so are mixed. A nurse explained that some clients say, “I don’t want to transmit this to other people; others would be like, ‘Whatever, I don’t care’.”

When names of partners who might be considered for self-contacting were not forthcoming, participants had to find ways to draw out such information and methods differed. One participant’s view of soliciting names was not unlike that of a detective uncovering clues. *“*The job of the buddy (counselor) is to dig up information as much as possible from the client.” He went on to explain that such information could be used to probe clients as to whom they might want to contact about shared exposure to the virus.

Building rapport was perceived as essential to persuading clients to identify and name their partners. Forging an empathetic connection, however, could require providers to proceed slowly and approach the client and topic with considerable caution. For one peer educator, this was not unlike dealing with a frightened animal:

Despite being a health care provider, if we want to approach them – it’s like we’re getting into a cat’s cage, we need to be like a cat so that we can just talk comfortably with them.

Others recommended building rapport by calling attention to a shared background. For example, one peer educator explained that during HIV counseling he purposively discloses to male clients who have sex with men that he does too. He explained, “I just open up about my own situation to speed up the process.” Another described the successful tactic of employing female-to-female patient/client gender-matching to open conversations with women about HIV exposure.

In addition to rapport, correct timing was seen as critical. A nurse warned that introducing partner services too early in the counseling process might jeopardize efforts to build rapport and undermine long-term relationships that would facilitate APN acceptance. Drawing on her HIV counseling experience to envision APN contact solicitation, she went on to explain, “If we suddenly aim for the partner, the trust that we have been building gradually can disappear in a moment.” Another HIV counselor remarked, “Clients are usually not yet open in the first or second meeting. They sometimes start to open up in the second or third meeting, and sometimes it takes more than three meetings.”

Maintaining provider–client confidentiality also was seen as a crucial APN element. To identify at-risk partners, clients would need to share information that might be embarrassing, stigmatizing or even legally self-incriminating. Consequently, it would be important for clients to feel that APN sessions were shrouded in professional secrecy. One participant explained, “You can’t be a “bucket mouth” [mulut ember] in spilling to others what a client has said. Another participant reported that she implicitly reassured clients during routine HIV counselling sessions that:

Everything that you share with me is only between you, the doctor, the nurse, and God. No one else would know. We will not disclose any single thing that you share with us, except on medical or legal grounds.

Maintaining provider/client confidentiality also could mean conversing within a conducive environment where others could not overhear. One nurse explained that to preserve privacy you would “have to talk to the patients in a very confidential way, in a private room, not at the patient’s bedside that is only covered with a curtain.”

While participants agreed with the importance of tracing partners to warn of possible exposure, they also strongly agreed that not every partner needed to be identified or told. They firmly held to the belief that clients have the right not to name or to disclose being HIV positive if their personal circumstance did not pose an HIV threat to others. One HIV service provider explained:

If the client is adherent to therapy and has no risk to transmit infection, we need to respect his right not to disclose his status. It is different if the client is an active drug user, is not taking ARVs [antiretrovirals], and suddenly is about to get married. That’s when we need to intervene.”

In instances where the client might not be able or willing to notify a partner or no one might be harmed, providers reported that they would need to weigh their role in soliciting names for public good against honoring clients’ right not to tell.

### Locating partners

Finding partners once they are named would pose a new set of potential APN challenges according to participants. Locating a partner could be relatively easy if the client and partner were closely related or communicated regularly. In contrast, tracing a casual partner with whom the client had little to no contact post-exposure could prove formidable. Having multiple casual partners would escalate the difficulty, especially if it involved a chain of exposure. A participant with HIV counseling experience remarked about his male clients whose partners include other men:

One person can meet another person, and another, and so on. They meet through dating apps. Maybe after they meet, they’ll just lose contact. So, it’s difficult.

The caseloads of many of the focus group peer educators and HIV counselors included individuals of both genders who exchange sex for money. Participants strongly agreed that locating commercial partners would prove tough. Due to stigma and legal complications, so could finding partners exposed through sharing drug equipment or sexual behaviors with someone of the same sex. Such behaviors often are conducted in secret with little personal information exchanged that later would permit HIV notification.

### Partner notification

Participants said that deciding to inform a partner of HIV exposure can be exceedingly stressful for some clients. Fear of harsh consequences was not uncommon, and participants offered numerous examples of clients whom they had HIV counseled who refused or shied away from disclosing the information themselves. Yet, they also noted that other clients appear willing and might possibly prefer to self-disclose over other methods. A positive feature of APN is that it offers three options from which clients can select in informing their at-risk partners, and not all partners need to be notified using the same method.

#### Self-disclosure notification

Because informing others of being HIV-positive can go both well or badly no matter how it is disclosed, participants perceived that it was ethically important to warn or remind clients of the possibility that revealing their status could have negative consequences. One peer educator regularly queried his clients about their personal readiness to disclose:

Are you sure you want to open up about your status? Because once you open up to someone, that person will never forget. So, make sure that when you disclose your status, you have dealt with yourself. Then you can deal with someone else. If you have not befriended your status, you better not disclose it to other people.

Another participant added:

I always say that: “if you disclose your status, you have to be ready for two things. The first thing is to lose, and the second thing is to be left. So, you must be ready to lose and to be left. Are you ready? If you are, I will help you. But if you’re not ready yet, we’ll just wait until you are. Until you are totally sure that you have dealt and made peace with yourself, you must think carefully about disclosure’s positive and negative impacts on your relationship.”

Pregnant women appeared particularly susceptible to worries as to the effects of disclosure on their relationships, especially when informing a male partner. An outreach worker repeated the words of a female client, “If I disclose my status while I’m pregnant, then he’d leave me. Who will take care of me?”.

Not knowing what to advise an expectant mother facing such fears, the participant went on to explain his view:

If they want to disclose it later after the child is born, let it be. Maybe they also have their strategy. But if they had to disclose during their pregnancy, that concerns my sense of humanity.

Other participants were quick to add that many clients, and not just partnered and pregnant women, were subject to fears of loss and rejection should their positive HIV status become known. “They’re afraid of being left,” said one peer educator. “The fear that the partner would leave is especially strong.”

Unfortunately, such fears seemed justified for both genders when the revelation of having contracted HIV treads on already shaky personal ground. Participants reported that not all HIV clients have families or significant others who accept their lifestyles, drug use, and/or other societally defined immoral behavior. When coupled with HIV, such disapproval might result in shunning, abandonment, or being forced out of partner or family relationships. Conversely, some clients were seen to proactively adopt withdrawal or social distancing tactics to avoid such conflict.

Even when clients were sufficiently coached and ready to notify others, it was the recipient of the information who might prove unprepared to hear the disclosure. The threat of shared exposure to a serious illness could negatively color partner relationships, while misinformation about how HIV is transmitted might worry family and friends unrealistically about possible contagion. Consequently, coaching clients in how to conduct self-disclosure and impart correct information was seen to be an important part of APN.

Drawing on their HIV counseling experience, providers gave examples of tutoring clients in how and what to say when first disclosing their HIV status. Gradual disclosure followed by incremental addition of further details was commonly recommended as was carefully crafting the message. As one peer educator explained, the client cannot abruptly say, “I’m HIV-positive. The person living with HIV needs to open the discussion between themselves and their partners smoothly.”

When direct methods failed or seemed too onerous, participants recommend using indirect methods. A peer educator recounted how a client whom he counseled persuaded his mother to tell his spouse for him. A nurse suggested that reluctant clients can use printed HIV information:

You can bring home a brochure. That’s a strategy I have used with patients. Bring these brochures. Let your wife read them or put them out on a table. When she asks you about it, that’s when you can open-up a discussion about your status.

Another suggestion was to leave HIV medication out where it easily could be found. Having indirectly forced an initial conversation, a fuller discussion could ensue once the client’s status was out in the open.

#### Provider assisted notification

Provider assisted notification involves a client choosing to have a trained provider deliver the news, typically without revealing the identity of the index person. This method demands that the notifier approach a stranger with the unpleasant information of exposure to a serious virus. Telephone contact is commonly used by HIV health providers to break the news but was considered tricky in a country with a high number of telephone scams. The providers predicted that a health notifier’s call might be mistaken as fraudulent:

The challenge would be great in Indonesian culture. People will ask, “Who gave you my number? Why am I being contacted? I haven’t done anything!”

Meanwhile, participants worried that visits by a health notifier to a partner’s home, office, or favorite hangout might not go unnoticed in Indonesia’s tight-knit neighborhoods. Keeping such discussions confidential might prove especially difficult in homes where partners and their extended families live in close physical proximity.

In making a notification, participants recounted the difficulties that they likely would face as messengers in conveying unwelcome HIV information:

If we just come visit the clients’ partners and tell them, “Your husband is positive,” Ouch! They will “throw a cooking pot” [melempar panci] at us! I personally would rather encourage them [the clients] to self-disclose.

Even participants with considerable street outreach experience in approaching strangers about HIV prevention flinched at the prospect of contacting partners in the privacy of their home with sensitive and potentially upsetting information.

Contacting partners of men who have sex with men was seen as especially challenging. In linking clients and their partners to HIV through same-sex behavior, a notification could inadvertently become public and place both men at risk for harmful social and legal repercussions. Another provider remarked about the special notification challenges likely with older adults. He explained that “they are less open [than younger clients], so it would be hard to find out the chain of transmission.” Female partners also were considered potentially hard to approach for partner notification, especially if they resided in a multi-generational family unit where maintaining confidentiality was complicated by proximity. Aware of Indonesia’s long history of societal paternalism, one provider with contact tracing experience explained:

If the client lives in the in-law’s house. That can be tough. It is much easier when the index patient is the husband because husbands have all the rights. But when it is the wife who is diagnosed first, that can be tough.”

Participants agreed to feeling ill prepared to facilitate disclosures that involved revelations of infidelity, same-sex relationships, drug use, or other behavior considered taboo. The thought that their actions could result in dissolution of a marriage or other partnership was especially troubling. “We’re afraid that if we encourage clients to disclose to their [marital] partner and they later separate, in the end we’d be blamed for it.” They also worried about the professional ethics of delivering notifications that could result in such outcomes.

Participants pondered if unmarried clients or those estranged from their families or partners would perceive APN services as potentially useful. A prison nurse described the plight of HIV-positive incarcerated men whom she counsels:

Most of the prisoners are the ‘lost boys.’ They don’t have family. They lived on the street, or they say the family lives far away in the village. Only one or two inmates ever gave us a chance to explain to their family. The rest were the lost boys.

Of course, prisoners are not the only clients who are estranged from their family or community of birth. The participants’ caseloads include rural, unmarried youth of both genders who flock to the city for excitement, employment, or to avoid the prying eyes of family members. Testing HIV-positive could prove hard for someone to self-admit to a family who was left behind.

#### Dual notification

Dual notification teams the provider and client in planning and delivering a notification. A major challenge to dual notification lies in finding a way to invite a partner to attend an APN session without explaining why. One participant relayed what a client told him in this regard:

My challenge is that if I want my wife to come with me to an AIDS NGO, what should I tell her? What should I say about it? [She would ask] what kind of place is it? I would find it hard to say that this is the place to do an HIV test.”

The group’s recommendation was that clients could inform their partner that a joint appointment had been scheduled to discuss a health problem. No further group advice was offered, however, as to what clients might say if asked why an appointment was needed. They did agree, however, that providers should never mention HIV when making initial contact or requesting partners to see them for a health reason.

From a provider’s perspective, APN dual notification offers the advantage of saving them from the challenges and full burden of informing a stranger about HIV exposure. Although the provider and client form a team in telling the partner, the final decision-making and aftermath of disclosure in APN rests primarily on the informer and informed. As one participant envisions how APN couple counseling would work:

The patient holds the central role, while the counselor’s role is to explain what they could do next. The counselor can describe the possibilities, but it is the couple who makes the decision and not the counselor as to whether they should continue their relationship or do something else. The patient should be told that this is something for you and your partner to decide. My job is to provide the correct and appropriate information.

Despite APN’s promotion of client-driven services, participants’ remarks indicate keen awareness that neither providers or clients have total control over the notification process or its outcome. Organizational culture and its socio-economic environment also influence the APN process and its results.

### Organizational and legal contingencies

Focus group participants were united in maintaining that organizational contingencies could both facilitate and hinder the delivery of effective APN services. They perceived a wide gap between the ambitions of those who want to develop APN and the reality of implementing it in many Indonesian health care settings. In discussing these gaps, four themes emerged.

First, participants were aware of resource shortages in the Indonesian healthcare system that could limit successful adoption of APN services. One health care counselor described the scarcities of the rural prison where she worked:

Maybe it [APN] would work in prisons in the city where both counseling and treatment are available. But in the prisons in smaller towns, we can only do counseling. My prison is in the middle of a jungle. We cannot get HIV medication. Our local community health center and local hospital don’t have it either. So, we are just confused. We have no idea what to do with patients.

Other participants reported similar shortages where they were employed.

Second, front-line staff in many clinical and NGO settings were thought to lack the knowledge, skills, and professional confidence needed to counsel patients effectively about HIV or APN:

Several times when I asked my team to educate patients about HIV, they were hesitant to do so because of being emptyheaded [kepala kosong]. They said, I’m afraid that I won’t be able to answer questions correctly if the patient asks me.

While not necessarily sharing this dim view of health care staff, group consensus held that, “All professionals, especially the doctors and nurses who communicate with patients every day, need training in breaking bad news.”

Third, merely offering relevant training was perceived as inadequate to ensuring a capable cadre of APN staff. Participants remarked that the constant staff turn-over common to many Indonesian health care settings could reduce the number of trained providers over time:

The problem is not that providers aren’t sufficiently trained, they are. The problem is with the structure of the health care system. At the province level, human resources can change every three months including a change in the head of the community health center. And staff who have been trained don’t necessarily transfer their knowledge to their successor. That becomes a problem. The second problem is that once someone is hired to deal with HIV counseling at a polyclinic, that person also may be assigned to do many other things as well. So they are not focused solely on providing HIV services.

Consequently, participants opined that provisions would need to be made within the health care system and at the local level to train new APN staff as they were hired.

Fourth, participants were concerned that APN might not be equitably delivered across medical sites and to all clients. Professional experience convinced them that negative attitudes and stigma toward people with HIV persisted within healthcare settings and among medical staff. They told of having encountered health care providers seemingly unable to counsel people whose lifestyle choices differed from their own. They also worried that notification of more challenging cases of HIV exposure might hinge on the whims or biases of individual medical providers. In such instances, providers might create their own rules for these services, selectively offer APN services to some clients but not others, conduct notifications without the client’s permission, and possibly breach client confidentiality.

Finally, uncertainty as to Indonesian law governing disclosure of patient information posed a potential stumbling block for many participants in contemplating APN. For example, a nurse questioned if discussing a client’s private or secretive behavior in the presence of a third party would be legal:

Nowadays, the trend at the inpatient units is to have more MSM patients. But, the MSM patients at the inpatient wards usually come with their wives. Then it’s easier for us to do notification since the wife is the legal partner, right? But if accompanied by a friend and we don’t know anything about the wife, that’s the challenging case.

Another participant warned that programs attempting to launch APN would first “need to have a solid ground on the country’s regulations” to protect the rights of clients, their partners, and the health care providers who notify them.

## Discussion

Health providers in this study agreed that APN services seemed worth implementing and potentially could be integrated successfully into Indonesia’s existing HIV care. They saw dual or provider-assisted partner notification as appealing options for clients preferring not to notify partners of HIV exposure themselves. Nonetheless, successful implementation of a new modality in real-world health settings is enhanced when providers and stakeholders view the evidence for its adoption as credible, sufficient resources are available, the context is favorable, and enabling mechanisms exist to facilitate implementation [14]. Providers in this study doubted that all these conditions fully exist within Indonesia’s current health care system at the level needed for APN’s widespread integration.

The process of locating partners was cited as posing a particularly daunting challenge, especially when clients knew little personal information about the partner. Although not mentioned by informants in this study, internet-based programs to assist HIV partner notification have gained increased popularity elsewhere and endorsement by the U.S. Centers for Disease Control and Prevention [31]. Their use in reaching partners has proved effective in several settings [32–35]. More research is warranted to determine how best to implement them ethically and safely within APN [36].

Clients and notification circumstances differ as the group discussions made clear, and providers in this study perceived that successful APN services must consider that some clients are more vulnerable than others to negative disclosure outcomes. Mindful that partner notification could reveal previously secretive or illegal behavior unknown to others, they cautioned that a client’s revelation of an HIV positive status could expose otherwise clandestine illegal or culturally defined “immoral” behavior. For example, some unknown number of Indonesian MSM conceal their same sex behavior to avoid the stigma and social condemnation that it can engender [37] and some conform to societal and family expectations to marry a woman [23]. HIV notification inadvertently could make this private information public. Women also were seen as vulnerable to negative consequences based on Indonesian social mores. Remnants of Indonesia’s patriarchal belief system, dictating women’s subordination to and dependence on a male partner, continue to influence its culture today [20]. Women who are diagnosed as HIV-positive can find themselves subject to moral judgment and social rejection based on cultural and religious beliefs that “good women” don’t engage in behavior resulting in HIV [38].

Successful adoption of a new health care modality depends, in part, on providers’ comfort with delivering it [14]. Even with client permission, the providers were not always in agreement or certain as to the ethics of contacting and possibly intruding on the privacy of a partner when making a notification. Possibly, they may have over worried. Despite concern in the literature and among providers that partner notification can result in physical retaliation or social harm to the index person from those who are notified, an extensive meta-analysis of findings from multiple research studies across 8 countries found few reports of such occurrences [8]. Some partners even express gratitude for the information and opportunity that notification provides to make good health care decisions [39]. Yet, our focus group participants’ examples of stigma and other negative consequences for Indonesian women, MSM and others in disclosing their HIV status or having it become public suggest the need for research examining the influence of differing cultural, religious, and contextual situations on the outcomes of partner notification itself rather than focusing primarily on the method, APN or otherwise, through which notification occurs.

Often unrecognized or not mentioned in discussions of HIV service delivery is that some health care providers experience their duties as stressful and emotionally demanding [40]. Despite highly endorsing the concept, providers in our study called attention to their own uncertainty as to APN’s feasibility, their potential guilt at orchestrating a notification that ends badly, and qualms as to when notification was even needed or legally possible. These findings suggest that to avoid undue provider stress and possibly job “burn out,” organizations need to recognize, acknowledge, and address the challenges that APN providers face [41]. They also need to institute policies, realistic job expectations, and access to counseling and other forms of social support if needed.

Participants pointed to shortages in HIV medicine, staffing, and other resources as barriers to successful implementation of APN in some Indonesian settings and geographic areas. Their concern raises an important question: What is the benefit to expanding APN services in settings where the benefits of HIV treatment are not widely nor equitably shared? This quandary echoes one debated early in the AIDS epidemic about the benefits of informing people of having contracted HIV prior to the development of effective treatments to assist their survival. While the rationale for not testing possibly can be argued, so can the reasons for delivering APN and offering HIV testing despite such shortages. Even without HIV treatment, individuals who know their status can protect their partners against infection, openly garner the social support that they might need, and take steps to avoid acquiring preventable co-infections that would accelerate and worsen their HIV condition. Moreover, offering exposed partners the opportunity to accept or decline testing places the power to make this critical health decision directly in the hands of those affected.

Our findings provide an insightful and highly useful window through which to view many of the challenges, barriers, and facilitators that experienced health care professionals perceive are likely to arise when encouraging and implementing APN services. Successful implementation of APN also requires investigation into its acceptability among all HIV stakeholders including clients, partners, administrators, family, and community members. To this end, we refer you to the excellent study conducted in Indonesia [21] that explores APN from the added vantage of clients and the general population.

Insights to four conditions that must be met in successfully implementing APN services emerged from the group discussions. First, building a workforce of able APN providers requires organizational investment in providing comprehensive APN training, including periodic in-service sessions, across the model’s many service steps and functional components. Core curricula should emphasize and reinforce respect for all HIV clients irrespective of lifestyle choices or preferences as to whom and how to tell a partner about HIV exposure. Such training needs to raise awareness and provide the guidance needed for providers to comfortably, fairly, and effectively tailor APN services to match clients’ individual needs and preferences. Education in best notification practices, including information about the country’s laws and policies governing health information disclosure, was seen as mandatory. Realistic job descriptions and written guidelines for APN providers should accompany this training but be sufficiently flexible to address the needs and safety of even the most vulnerable client [42].

Second, as our informants reported, even health care workers are not immune to holding stigmatizing attitudes and enacted discrimination toward people with HIV including in Indonesia [24]. Based on a meta-analysis of the health care stigma literature [43], four strategies appear to work in reducing provider stigma: educating providers about the condition or about stigma and its effects on health; “skills-building activities” that allow healthcare providers to develop the necessary skills to work with members of a stigmatized group; “participatory learning” in which providers actively engage in the intervention; and either natural or arranged contact that breaks down stereotypes and prejudice and affords health care workers the opportunity to build human empathy toward the stigmatized group.

Third, all participants agreed that soliciting partners’ names and delivering effective APN services requires having built rapport with each client within a context of confidentiality and trust. Clients need to know and believe that disclosure within a provider/client session is private and confidential. Besides discussing APN’s benefits for both clients and partners, providers also should be forthcoming about the risks of disclosure and, when feasible and appropriate, suggest possible strategies to help ameliorate them. Security measures need to be in place to protect sensitive data and patient health records [44].

## Limitations of the study

The study’s health care providers were sampled through their professional networks using internet and word-of-mouth publicity. Consequently, the study’s results may not generalize to health care providers whom these methods failed to reach or who chose not to participate. Also, providers who were familiar with APN and its potential benefits may have been more likely to volunteer than those unfamiliar with APN or who judged it negatively. In addition, our focus groups were composed of providers with varying partner notification experience who were drawn from health occupations of differing professional status. Such variation can affect the power dynamics of the group with some participants feeling freer to speak than others [45]. On the other hand, such diversity also can result in a variety of views being raised for discussion that might not have emerged with a more homogeneous group.

## Conclusions

Mounting evidence from multiple countries testifies to the success of assisted partner notification (APN) in safely and significantly increasing the uptake of HIV testing among the partners of HIV-positive clients. Yet achieving this success is not without some degree of problems and challenge. The findings and insights into APN gained from this study add to the growing body of research needed to guide APN’s effective adoption within countries and settings where it will be newly introduced and then maintained.

## Supplementary Information

**Additional file 1.**

## Data Availability

Data requests referencing protocol #2018–0754 may be sent to the Director of Research Facilitation at University of Illinois Chicago, College of Nursing, Susan Littau (slittau@uic.edu).

## Abbreviations

APN: Assisted partner notification
LGBT: Lesbian, gay, bisexual, transgender
MSM: Men who have sex with men
NGO: Nongovernment organization
PARIHS: The Promoting Action on Research Implementation in Health Sciences
