# Supplementary material for: A qualitative study of healthcare providers’ attitudes toward assisted partner notification for people with HIV in Indonesia
Source: BMC Health Serv Res. 2023 Jan 24;23:71. doi: 10.1186/s12913-022-08943-x (PMC9872286; doi:10.1186/s12913-022-08943-x)
Supplement: Supplementary file 1 — Additional file 1. [file 12913_2022_8943_MOESM1_ESM.docx]

**APPENDIX 1**

**Focus Group Discussion Guide**

Thank you for agreeing to participate in this discussion. The purpose of this discussion is to help us (researchers) understand whether nurses in Indonesia are comfortable and ready to conduct HIV partner notification. Assisted HIV partner notification services refers to when consenting HIV-positive clients are assisted by a trained healthcare provider to disclose their HIV status or to anonymously notify their sex and/or drug injecting partners of their potential exposure to HIV infection. The provider then offers HIV testing to these partner(s). Healthcare providers may notify partners by phone or in person at the person’s home or in a healthcare facility.

HIV diagnosis is the first step to treatment. Many people with HIV, however, are unaware of their HIV status. With assisted partner notification, also called APN, trained health notifiers assist people with HIV to notify their partners of possible exposure to the virus and offer them testing. APN has proven to be safe and effective in several studies and WHO recommends APN to increase HIV testing among partners of people diagnosed with HIV.

Today, we are interested in learning about your experiences in helping clients who want to notify partners and your thoughts toward implementing APN with clients in the settings where you work.

We would like everyone to have a chance to talk. There are no right or wrong answers. Also, whatever is shared in this discussion should remain confidential and not be discussed outside the group. Finally, researchers will protect the information that you share today and your responses will not identify you as a participant in research. With your permission, we will digitally record this discussion. We will also take notes to summarize the main points of discussions. Are there any questions before we begin?

**A. Health Care Provider Roles and Responsibilities**

- Describe your roles and responsibilities as they relate to HIV prevention at your agency?
- How would you characterize your work and your relationship with clients?
- What are some of the strategies that you use to maintain those relationships?
- What problems or issues do clients most often ask for help with?
- What is your role in helping them to address such requests?
- Can you discuss the roles and responsibilities of [health care providers or peer educators] in assisting HIV-positive clients to disclose their HIV status or contact their partners for HIV testing. Can you give an example of how this is done at your agency?
- How might [health care providers or peer educators] deliver such sensitive information?
- What types of training might be needed for [health care providers or peer educators] to assist clients in notifying their partners, including notification using APN
- How is contact tracing and notification for HIV different from contact tracing for other diseases or conditions?

**B. Professional Experience**

- How would you describe your colleagues [health care providers or peer educators] comfort or experience in discussing or giving health advice to clients about HIV preventing HIV transmission.
- How would you or your professional colleagues feel about calling someone you don’t know to tell them that they may have been exposed to the HIV virus?
- If the partner could not be reached by phone, how comfortable would you or your colleagues be going to the person’s home?
- Would it make any difference if the person being notified was a man or a woman?
- Would it make any difference if the person being notified was assumed to be involved in same-sex or illegal drug injection behaviors?
- How confident or comfortable are you in explaining a positive HIV test result – that is, if the test indicated that the person was infected with HIV?
- How confident do you feel when talking to someone who is recently diagnosed with HIV about accessing follow-up services?
- What additional skills or information would be helpful to be able to assist clients who want to notify their partners?
- What changes may be helpful in your workplace so that you are able to assist client who want to notify a partner?
- Are there times when you have been concerned about your own personal safety or health safety in the workplace? Which activities have caused you to be concerned?

**C. Interprofessional collaboration**

I want to learn about your experiences working with colleagues from other professions or disciplines at your agency. Specifically, I’m interested in hearing about how roles and responsibilities are assigned and what you see

- Can you comment on how the knowledge and skills of other health care providers or HIV service providers contributes to the mission or work at your agency?
- What are the strengths of health care or HIV service providers in assisting clients to name or consent to notify their partners?
- How trusted or well-regarded are providers by clients at your agency?
- Are some partner notification activities more suitable for a peer educator? Which partner notification activities might be more suitable for a nurse?

**D. Community Perceptions**

I want to learn from you about how people in the community might respond to APN activities. For example, neighbors or other “third parties” who might be directly or indirectly aware of HIV notifications that occur in their home, workplace, or neighborhood.

- What concerns, if any, might you have about maintaining client or partner confidentiality in your practice settings
- What concerns, if any, might you have about maintaining client or partner confidentiality when you first approach partners at home or in the community?
- What strategies seem to work best for assisting clients who wish to maintain confidentiality?
- What information do you offer others who may inquire about the reasons why they are being contacted?

**E. Deservingness of Care**

- To what extent do people with HIV face neglect from their family or rejection by their peers? Could you share an example of time when you heard of this happening?
- To what extent do people with HIV experience neglect or unfair treatment in health care? Do you see examples of unequal treatment for people with or at-risk for HIV in your work settings?
- In your experience, do health care providers create a welcoming environment for the client?
- What sorts of feedback do you receive from clients about the services that your agency provides?

**F. Duty to Inform**

- I would like to ask you to consider instances where clients or healthcare providers might disagree about whether or not a partner should be notified or how that should happen.
- Can you describe a situation where you have felt conflicted about whether or not to share a client’s HIV status or diagnosis with or without their permission?
- Can you describe a situation where you felt compelled to share information about a client’s HIV status?
- Can you think of a situation where a professional or occupational duty to inform might supersede a patient’s right to confidentiality?
